# Total body irradiation versus busulfan based intermediate intensity conditioning for stem cell transplantation in ALL patients >45 years—a registry-based study by the Acute Leukemia Working Party of the EBMT

**DOI:** 10.1038/s41409-023-01966-w

**Published:** 2023-05-05

**Authors:** Klaus Hirschbühl, Myriam Labopin, Emmanuelle Polge, Didier Blaise, Jean Henri Bourhis, Gerard Socié, Edouard Forcade, Ibrahim Yakoub-Agha, Hélène Labussière-Wallet, Wolfgang Bethge, Patrice Chevallier, Sarah Bonnet, Matthias Stelljes, Alexandros Spyridonidis, Zinaida Peric, Eolia Brissot, Bipin Savani, Sebastian Giebel, Christoph Schmid, Fabio Ciceri, Arnon Nagler, Mohamad Mohty

**Affiliations:** 1 Augsburg University Hospital and Medical Faculty, Augsburg, Germany; 2 EBMT Statistical Unit, Sorbonne Université, INSERM UMR-S 938, CRSA, Service d’hématologie et Thérapie Cellulaire, AP-HP, Hôpital Saint-Antoine, 75 012 Paris, France; 3 Programme de Transplantation & Therapie Cellulaire, Centre de Recherche en Cancérologie de Marseille, Institut Paoli Calmettes, Marseille, France; 4 Department of Hematology, Gustave Roussy Cancer Campus BMT Service, Villejuif, France; 5 Department of Hematology – BMT, Hopital St. Louis, Paris, France; 6 CHU Bordeaux, Hôpital Haut-leveque, Pessac, France; 7 CHU de Lille LIRIC, INSERM U995, Université de Lille, Lille, France; 8 Centre Hospitalier Lyon Sud, Service Hematologie, Lyon, France; 9 Universitaet Tuebingen, Medizinische Klinik, Abteilung II, Tuebingen, Germany; 10 CHU Nantes Department D’Hematologie, Nantes, France; 11 Département d’Hématologie Clinique, CHU Montpellier, Hôpital Saint Eloi, Montpellier, France; 12 Department of Medicine A—Hematology, Hemostaseology, Oncology, Pulmonology, University Hospital Muenster, 48149 Munster, Germany; 13 Department of Internal Medicine, BMT Unit and CBMDP Donor Center, University of Patras, Patras, Greece; 14 Zagreb School of Medicine, University Hospital Centre Zagreb, Zagreb, Croatia; 15 APHP, Hôpital Saint Antoine, Service d’Hématologie Clinique et de Thérapie Cellulaire, Paris, France; 16 Division of Hematology and Oncology, Vanderbilt University, Nashville, TN USA; 17 Maria Sklodowska-Curie National Research Institute of Oncology, Gliwice, Poland; 18 IRCCS Ospedale San Raffaele, University Vita-Salute San Raffaele, Milan, Italy; 19 Division of Hematology and Bone Marrow Transplantation, The Chaim Sheba Medical Center, Tel-Hashomer, Ramat-Gan, Israel

**Keywords:** Stem-cell therapies, Stem-cell research

## Abstract

Allogeneic hematopoietic cell transplantation is a potentially curative treatment in high-risk acute lymphoblastic leukemia (ALL). Conditioning regimens based on ≥12 Gray total body irradiation (TBI) represent the current standard in patients ≤45 years, whereas elderly patients frequently receive intermediate intensity conditioning (IIC) to reduce toxicity. To evaluate the role of TBI as a backbone of IIC in ALL, a retrospective, registry-based study included patients >45 years transplanted from matched donors in first complete remission, who had received either fludarabine/TBI 8 Gy (FluTBI8, *n* = 262), or the most popular, irradiation-free alternative fludarabine/busulfan, comprising busulfan 6.4 mg/kg (FluBu6.4, *n* = 188) or 9.6 mg/kg (FluBu9.6, *n* = 51). At two years, overall survival (OS) was 68.5%, 57%, and 62.2%, leukemia-free survival (LFS) was 58%, 42.7%, and 45%, relapse incidence (RI) was 27.2%, 40%, and 30.9%, and non-relapse-mortality (NRM) was 23.1%, 20.7%, and 26.8% for patients receiving FluTBI8Gy, FluBu6.4, and FluBu9.6, respectively. In multivariate analysis, the risk of NRM, acute and chronic graft-versus-host disease was not influenced by conditioning. However, RI was higher after FluBu6.4 (hazard ratio [HR] [95% CI]: 1.85 [1.16–2.95]), and LFS was lower after both FluBu6.4 (HR: 1.56 [1.09–2.23]) and FluBu9.6 (HR: 1.63 [1.02–2.58]) as compared to FluTBI8. Although only resulting in a non-significant advantage in OS, this observation indicates a stronger anti-leukemic efficacy of TBI-based intermediate intensity conditioning.

## Introduction

In high-risk adult acute lymphoblastic leukemia (ALL) allogeneic hematopoietic cell transplantation (alloHCT) represents the treatment option with the highest potential for cure. Hence, alloHCT has been increasingly used over the years [1, 2], resulting in an age-dependent 5-year overall survival (OS) of between 41 and 66% [3–5]. For patients up to about 45 years of age, total body irradiation (TBI) ≥ 12 Gray (Gy) represents the standard backbone for conditioning, which is applied in combination with different chemotherapeutic agents (etoposide, cyclophosphamide, fludarabine) [6–8]. However, these classical myeloablative conditioning regimens are accompanied by a relevant toxicity in older patients [9, 10]. Therefore, dose-adapted or intermediate intensity conditioning (IIC) regimens such as fludarabine/TBI 8 Gy (FluTBI8) are frequently used in patients over the age of 45 years. These regimens are still myeloablative but contain reduced dosages of classical conditioning elements. Considering patients’ comorbidities, availability of irradiation facilities and patients’ or physicians’ preference, irradiation-free alternatives have been developed, with fludarabine/busulfan being the most popular combination. In Germany the recommended regimen is fludarabine/busulfan 6.4 mg/kg (FluBu6.4) [11], whereas in other countries fludarabine/busulfan 9.6 mg/kg (FluBu9.6) is used more frequently. While several studies have investigated the issue of the reduced/IIC regimen in ALL [12–17], no direct comparison has addressed the role of TBI among IIC regimens, both with respect to efficacy and toxicity.

## Materials and methods

### Study design and cohort

A retrospective, European Society of Blood and Marrow Transplantation (EBMT) registry-based study was performed. The EBMT is a non-profit, scientific society representing more than 600 transplant centers mainly in Europe, that are required to report to the registry all consecutive stem cell transplantations including follow-up once a year. Data are managed in a central database with internet access, in which each EBMT center is represented. Annual audits are performed to verify data accuracy. EBMT centers commit to obtain informed consent according to the local regulations applicable at the time of transplantation in order to report pseudonymized data to the EBMT. Inclusion criteria were: (1) Age >45 years, (2) allogeneic hematopoietic cell transplant (alloHCT) from matched sibling or matched unrelated donors between 2005 and 2020 for ALL in first complete remission, (3) conditioning with either FluTBI8, FluBu6.4 or FluBu9.6. The study was approved by the general assembly of the Acute Leukemia Working Party of the EBMT.

### Endpoints and definitions

Overall survival (OS) and leukemia free survival (LFS) were the major endpoints of interest. The cumulative incidence of non-relapse mortality (NRM) and relapse incidence (RI), as well as acute and chronic graft-versus-host disease (a/cGvHD) and GvHD-free, relapse-free survival (GRFS) were also analyzed. OS was defined as the interval from date of alloHCT to date of last follow-up (LFU) or date of death, regardless of cause. LFS was calculated as the interval between the date of alloHCT and death, relapse, or last follow-up. NRM was defined as death without previous relapse or progression, GRFS as survival from alloHCT without aGvHD grade III–IV, without severe cGvHD and without evidence of relapse. Acute and chronic GvHD were classified as previously described [18, 19]. Measurable residual disease (MRD) status was evaluated according to local standards, including *BCR::ABL* PCR, flow cytometry and individually identified IgHV or T-cell receptor rearrangements, and was included into the analysis as reported by the participating centers.

### Statistical analysis

Patient-, disease-, and transplant-related characteristics were compared using the chi-square or Fisher’s exact test for categorical variables, and the Wilcoxon test for continuous variables between the three conditioning regimens. The probabilities of OS, LFS, and GRFS were calculated using the Kaplan-Meier estimate. The probabilities of relapse incidence (RI), NRM, acute and chronic GVHD were estimated using cumulative incidence curves. For GVHD, death and relapse were considered competing events. Univariate analyses were performed using the log-rank test for OS, LFS, and GRFS, and Gray’s test for RI, NRM and GVHD. Multivariate analysis was performed using a Cox proportional-hazards model which included variables differing significantly (*p* < 0.05) between the groups, factors known to be associated with outcomes, plus a center frailty effect to take into account the heterogeneity across centers. For all comparisons, follow-up was censored at 2 years in order to take into account for the differences in follow-up between the 3 groups.

Results were expressed as the hazard ratio (HR) with the 95% confidence interval (95% CI). All tests were two-sided with a type 1 error rate fixed at 0.05. The Bonferroni correction was used to control the type I error when testing the differences among the three levels of the conditioning. Statistical analyses were performed with SPSS 25.0 (IBM Corp., Armonk, NY, USA) and R 4.0.2 (R Core Team (2020). R: A language and environment for statistical computing. R Foundation for Statistical Computing, Vienna, Austria. URL https://www.R-project.org/).

## Results

### Patients’ characteristics

In total, 501 patients were identified (Philadelphia chromosome negative (Ph-) B-ALL, *n* = 139; Ph+ B-ALL, *n* = 296; T-ALL, *n* = 66). Conditioning for alloHCT comprised FluTBI8 (*n* = 262), FluBu6.4 (*n* = 188) or FluBu9.6 (*n* = 51). Patient characteristics revealed imbalances among the three cohorts with respect to median age (*p* < 0.0001), ALL subtype (*p* = 0.025), HCT-CI (*p* = 0.0002), in-vivo T-cell depletion (*p* < 0.0001), CMV patient/donor serostatus (*p* = 0.01) and median year of transplant (*p* < 0.0001), whereas all other features were balanced. Median follow-up from transplant was 21, 53, and 32 months, *p* < 0.0001. Further information on patient- and transplant-related characteristics are shown in Table 1.

**Table 1:** Patient and transplant characteristics.

		FluTBI8 (*n* = 262)	FluBu6.4 (*n* = 188)	FluBu9.6 (*n* = 51)	*p*
Median FU (months)		21	53	32.3	<0.0001
Patient age (years)	median (min-max)	56 (45.4–76.1)	60.3 (45.1–72)	55.4 (45.9–65.6)	<0.0001
Year transplant	median (min-max)	2018 (2005–2020)	2014 (2007–2020)	2015 (2009–2020)	<0.0001
Diagnosis	Ph- B-ALL	76 (29%)	53 (28.2%)	10 (19.6%)	0.025
	Ph+ B-ALL	142 (54.2%)	121 (64.4%)	33 (64.7%)	
	T-ALL	44 (16.8%)	14 (7.4%)	8 (15.7%)	
Patient sex	male	144 (55%)	80 (42.6%)	26 (51%)	0.034
	female	118 (45%)	108 (57.4%)	25 (49%)	
KPS	<90	77 (29.4%)	61 (32.4%)	12 (23.5%)	0.45
	>=90	185 (70.6%)	127 (67.6%)	39 (76.5%)	
HCT-CI	HCT-CI = 0	103 (39.3%)	38 (20.2%)	22 (43.1%)	0.0002
	HCT-CI = 1/2	35 (13.4%)	27 (14.4%)	5 (9.8%)	
	HCT-CI > = 3	49 (18.7%)	35 (18.6%)	6 (11.8%)	
	missing	75 (28.6%)	88 (46.8%)	18 (35.3%)	
MRD pre HCT	MRD−	135 (51.5%)	75 (39.9%)	18 (35.3%)	0.008
	MRD+	73 (27.9%)	49 (26.1%)	19 (37.3%)	
	missing	54 (20.6%)	64 (34%)	14 (27.5%)	
Donor	MSD	110 (42%)	84 (44.7%)	21 (41.2%)	0.69
	UD 10/10	127 (48.5%)	80 (42.6%)	25 (49%)	
	UD 9/10	25 (9.5%)	24 (12.8%)	5 (9.8%)	
Donor sex	male	169 (64.5%)	101 (54%)	34 (68%)	0.045
	female	93 (35.5%)	86 (46%)	16 (32%)	
	missing	0	1	1	
f to m combination	no f to m	223 (85.1%)	154 (82.4%)	42 (82.4%)	0.7
	f to m	39 (14.9%)	33 (17.6%)	9 (17.6%)	
	missing	0	1	0	
Cell source	Bone Marrow	22 (8.4%)	6 (3.2%)	4 (7.8%)	0.052
	Peripheral Blood	240 (91.6%)	182 (96.8%)	47 (92.2%)	
Donor/Patient CMV	Not CMV−/−	176 (67.7%)	141 (77%)	42 (85.7%)	0.01
	CMV−/−	84 (32.3%)	42 (23%)	7 (14.3%)	
	missing	2	5	2	
PTCy	No PTCy	241 (93.8%)	178 (96.2%)	48 (94.1%)	0.47
	PTCy	16 (6.2%)	7 (3.8%)	3 (5.9%)	
	missing	5	3	0	
In vivo TCD	no in vivo TCD	96 (37.1%)	18 (9.6%)	10 (19.6%)	<0.0001
	in vivo TCD	163 (62.9%)	170 (90.4%)	41 (80.4%)	
	missing	3	0	0	

*FU* follow up, *KPS* Karnofsky performance status, *HCT-CI* hematopoietic cell transplantation-comorbidity index, *MRD* measurable residual disease, *f* female, *m* male, *CMV* cytomegalovirus, *PTCy* post-transplant cyclophosphamide, *TCD* T-cell depletion, *min* minimum, *max* maximum, *Ph* Philadelphia chromosome, *MSD* matched sibling donor, *UD* unrelated donor.

### Outcome

**Fig. 1:**
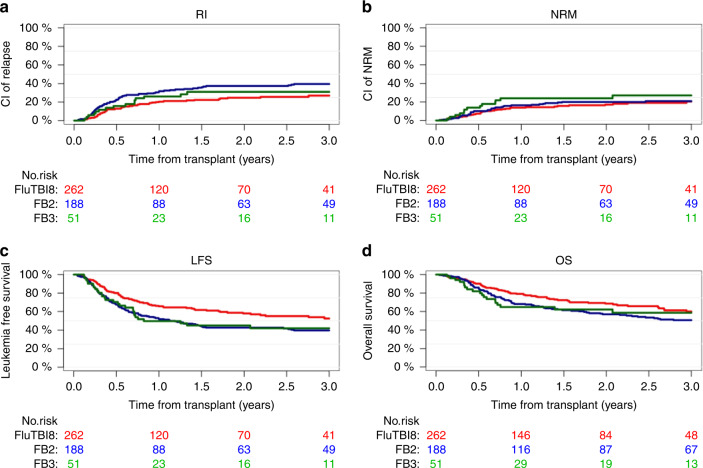
Comparison of FluTBI8, FluBu6.4 (FB2) and FluBu9.6 (FB3). Relapse incidence (**a**), non-relapse mortality (**b**), leukemia-free survival (**c**) and overall survival (**d**).

After conditioning with FluTBI8, FluBu6.4 or FluBu9.6, OS at two years from alloHCT was 68.5%, 57%, and 62.2% (*p* = 0.06). RI at two years was significantly different between the 3 groups: 24.7% among patients receiving FluTBI8, 37.3% and 30.9% in FluBu6.4 and FluBu9.6, respectively (*p* = 0.014), translating to a different LFS, being 58% after conditioning with TBI8Gy, 42.7%, and 45% after Bu-based conditioning (*p* = 0.003). Among the three groups, cumulative incidence of NRM at two years was not different (FluTBI8: 17.3%, FluBu6.4: 20.1%, and FluBu9.6: 24%, *p* = 0.38), and GRFS was 39.9%, 34.3%, and 40.1%, respectively (*p* = 0.29). Time-to-event outcomes are illustrated in Fig. 1 and Supplementary Table 1.

### GvHD and cause of death

The cumulative incidence of aGvHD grade II-IV did not vary among the groups being 26.3% for FluTBI8 and 26.3% and 29.2% for FluBu6.4 and FluBu9.6, respectively (*p* = 0.9). Acute GvHD grade III–IV was 9%, 8.8% and 8.2% for the three regimens (*p* = 0.97). Regarding chronic GVHD (limited and extensive), cumulative incidences were 45.7%, 33.2% and 31.1% (global *p* value = 0.018, but the differences were not confirmed in multivariate analysis). For extensive cGVHD, cumulative incidences were 23.5%, 15.9% and 10.9%, *p* = 0.07 (see Supplementary Tables 1, 2).

Leukemia relapse was the main cause of death in all three cohorts (51.7% of all fatalities), followed by GvHD (20.2%) and infections (15.7%). Patients receiving FluTBI8 showed the highest rate of infection-associated deaths (24.3% as compared to 15.8%/8.2% after FluBu9.6/FluBu6.4). The highest rate of death caused by GvHD was seen with FluBu9.6 (36.8%), while FluBu6.4 (23.5%) and FluTBI8 (12.2%) showed lower rates. Further details on other causes of death are shown in Table 2.

**Table 2:** Causes of death.

Cause of death	FluTBI8 (*n* = 77)	FluBu6.4 (*n* = 87)	FluBu9.6 (*n* = 19)	Overall (*n* = 183)
Hemorhage	1 (1.4%)	0 (0%)	0 (0%)	1 (0.6%)
VOD	0 (0%)	1 (1.2%)	1 (5.3%)	2 (1.1%)
Infection	18 (24.3%)	7 (8.2%)	3 (15.8%)	28 (15.7%)
GvHD	9 (12.2%)	20 (23.5%)	7 (36.8%)	36 (20.2%)
Original disease	35 (47.3%)	51 (60%)	6 (31.6%)	92 (51.7%)
Other malignancy	3 (4.1%)	0 (0%)	0 (0%)	3 (1.7%)
MOF	1 (1.4%)	1 (1.2%)	0 (0%)	2 (1.1%)
CNS toxicity	1 (1.4%)	1 (1.2%)	0 (0%)	2 (1.1%)
Other transplant related	2 (2.7%)	1 (1.2%)	0 (0%)	3 (1.7%)
Non HCT related	4 (5.4%)	3 (3.5%)	2 (10.5%)	9 (5.1%)
missing	3	2	0	5

*VOD* veno-occlusive disease, *GvHD* graft-versus-host disease, *MOF* multiple-organ failure, *CNS* central nervous system, *HCT* hematopoietic cell transplantation.

### Multivariate analysis

In comparison to FluTBI8, RI was significantly higher after FluBu6.4 (hazard ratio [HR] [95% confidence interval [CI]: 1.85 [1.16–2.95], *p* = 0.01), but not after FluBu9.6 (HR: 1.51 [0.82–2.77], *p* = 0.19). LFS was inferior after conditioning with both FluBu6.4 (HR: 1.56 [1.09–2.23], *p* = 0.014) and FluBu9.6 (HR: 1.63 [1.02–2.58], *p* = 0.039) as compared to FluTBI8. However, OS was not significantly different among the three subgroups. Risk of NRM, GRFS, aGvHD II–IV, aGvHD III–IV and both limited and extensive cGvHD were not influenced by conditioning type either.

Other factors significantly influencing outcomes were increasing age (per 10 years, no interaction between age and conditioning), Ph+ ALL subtype, and MRD status at start of conditioning for alloHCT: Age was associated with higher NRM and lower OS, LFS, and GRFS. Patients with Ph+ ALL had a significantly lower RI and a better OS, LFS, and GRFS, whereas MRD had a negative impact on RI, LFS and GRFS. Results of the multivariate analysis are shown in Table 3 and Supplementary Table 2.

**Table 3:** Multivariate analysis of risk factors on outcome parameters.

	RI		NRM		LFS		OS		GRFS	
	HR (95% CI)	*p* value	HR (95% CI)	*p* value	HR (95% CI)	*p* value	HR (95% CI)	*p* value	HR (95% CI)	*p* value
FluTBI8 (ref.)	1		1		1		1		1	
FluBu6.4	1.85 (1.16–2.95)	0.01	1.31 (0.72–2.36)	0.38	1.56 (1.09–2.23)	0.014	1.31 (0.84–2.04)	0.23	1.25 (0.92–1.72)	0.15
FluBu9.6	1.51 (0.82–2.77)	0.19	1.91 (0.89–4.1)	0.099	1.63 (1.02–2.58)	0.039	1.41 (0.79–2.54)	0.25	1.1 (0.72–1.7)	0.65
Age (per 10 y)	1.25 (0.9–1.74)	0.19	2.1 (1.36–3.24)	0.0008	1.53 (1.18–1.99)	0.002	1.82 (1.33–2.5)	0.0002	1.29 (1.03–1.61)	0.026
Ph neg B-ALL (ref.)	1		1		1		1		1	
Ph pos B-ALL	0.4 (0.26–0.62)	<0.0001	1.21 (0.64–2.27)	0.56	0.59 (0.42–0.83)	0.003	0.61 (0.4–0.93)	0.021	0.68 (0.5–0.91)	0.011
T-ALL	0.81 (0.47–1.42)	0.47	2.01 (0.89–4.53)	0.092	1.06 (0.67–1.66)	0.81	1.11 (0.66–1.89)	0.69	1.02 (0.68–1.54)	0.92
MRD− (ref.)	1		1		1		1		1	
MRD+	2.02 (1.31–3.11)	0.001	1.01 (0.57–1.8)	0.97	1.53 (1.09–2.14)	0.014	1.34 (0.88–2.04)	0.17	1.45 (1.07–1.95)	0.015
MRD unknown	1.1 (0.69–1.76)	0.68	1.04 (0.55–1.97)	0.9	1.06 (0.73–1.52)	0.77	1.25 (0.8–1.93)	0.33	1.09 (0.78–1.51)	0.62
Year of HCT	0.98 (0.93–1.04)	0.59	0.97 (0.9–1.04)	0.36	0.98 (0.94–1.02)	0.27	0.97 (0.92–1.03)	0.31	0.99 (0.95–1.03)	0.55
Female to Male	0.86 (0.54–1.38)	0.54	1.14 (0.6–2.17)	0.68	0.96 (0.66–1.39)	0.83	0.99 (0.63–1.54)	0.95	0.98 (0.7–1.36)	0.9
KPS > = 90	1.27 (0.86–1.87)	0.23	1.01 (0.6–1.69)	0.98	1.18 (0.87–1.6)	0.29	1.01 (0.7–1.46)	0.94	1.15 (0.88–1.51)	0.31
MSD (ref.)	1		1		1		1		1	
UD 10/10	0.67 (0.44–1.01)	0.059	1.34 (0.76–2.36)	0.31	0.86 (0.62–1.2)	0.37	0.82 (0.55–1.23)	0.34	0.94 (0.7–1.26)	0.67
UD 9/10	0.56 (0.29–1.08)	0.084	1.31 (0.61–2.82)	0.49	0.77 (0.48–1.26)	0.3	0.87 (0.49–1.53)	0.62	0.87 (0.57–1.33)	0.53
PB vs BM	0.88 (0.44–1.76)	0.71	0.97 (0.37–2.54)	0.95	0.91 (0.52–1.59)	0.74	0.69 (0.36–1.31)	0.26	0.93 (0.57–1.52)	0.77
in vivo TCD	0.85 (0.52–1.39)	0.52	0.61 (0.33–1.14)	0.12	0.74 (0.51–1.08)	0.12	0.84 (0.52–1.35)	0.48	0.73 (0.53–1.01)	0.06
Patient/Donor CMV−/− vs other	1.21 (0.82–1.81)	0.34	1.17 (0.68–2.03)	0.57	1.21 (0.88–1.67)	0.23	1.16 (0.78–1.7)	0.46	1.27 (0.96–1.67)	0.092
Centre (frailty)		0.92		0.19		0.91		0.25		0.29

*HR* hazard ratio, *CI* confidence interval, *ref*. reference, *y* year, *Ph* Philadelphia chromosome, *MRD* measurable residual disease, *HCT* hematopoietic cell transplantation, *KPS* Karnofsky performance status, *MSD* matched sibling donor, *UD* unrelated donor, *PB* peripheral blood, *BM* bone marrow, *TCD* T-cell depletion, *CMV* cytomegalovirus, *RI* relapse incidence, *NRM* non-relapse mortality, *LFS* leukemia-free survival, *OS* overall survival, *GRFS* graft-versus-host disease-free/relapse-free survival

## Discussion

To the best of our knowledge, this is the first analysis evaluating the role of TBI for ALL patients aged >45 years transplanted in first complete remission receiving an IIC regimen. The most frequently used radiation-free regimens (FluBu6.4 and FluBu9.6) have been used for comparison.

Among standard conditioning regimens in ALL, TBI containing regimens (usually comprising ≥12 Gy), were associated with better antileukemic efficacy as compared to irradiation-free protocols [8, 20, 21]. Hence, ≥12 Gy TBI-based regimens are regarded as standard for myeloablative conditioning for alloHCT in younger adults and children with ALL [11, 22, 23]. However, NRM was increased among patients >45 years of age receiving standard protocols [9, 10, 24]. Relevant toxicities have been described in particular for TBI compared to TBI-free conditioning regimens, with non-infectious pulmonary toxicity being a relevant issue in this context [25–28]. Therefore, dose adapted IIC regimens have been developed. In contrast to the observations made after standard conditioning, our comparison of IIC regimen did not show an increased toxicity of the TBI containing protocol as compared to busulfan-based regimen. Overall, NRM was 23.1% after TBI and 20.7% and 26.8% after FluBu6.4 and FluBu9.6 conditioning, respectively. With respect to the causes of death, lethal infectious complications were more frequent after TBI-based conditioning, which may be a consequence of an increase in mucosal toxicity as a possible cause for bacterial infections. Particular attention should therefore be paid to mucosal protection and early anti-microbiological intervention in patients receiving TBI. In contrast to data reported following standard conditioning protocols, non-infectious lung toxicity was not observed as a frequent cause of death among patients receiving FluTBI8. Overall, GvHD was a more frequent cause of death among patients receiving a busulfan-based regimen, although the cumulative incidence of severe a/cGvHD was not different.

With respect to efficacy, we found that FluTBI8 was associated with a lower RI than FluBu6.4, and a superior LFS (58% at two years) as compared to both FluBu cohorts (42.7%, HR: 1.56 [1.09–2.23] and 45%, HR: 1.63 [1.02–2.58]). This data suggests a superior antileukemic potential of intermediate dose TBI in comparison to chemotherapy-based conditioning. Similar to our data, a recent study comparing FluTBI8 to FluBu9.6 given before alloHCT in acute myeloid leukemia observed an improved LFS for patients <50 years receiving TBI [29]. In contrast, an earlier study from the ALWP of the EBMT in patients with ALL found no difference between FluBu6.4 and a conditioning containing TBI at a non-myeloablative dose of 2 Gy, underpinning that a minimal dosage of TBI that is necessary for an antileukemic effect [16]. Similarly, no difference was observed in a recent randomized phase III trial comparing standard dose busulfan to myeloablative TBI in younger patients with standard risk ALL [30]. On the other hand, 8 Gy might also represent an optimal upper dose for TBI, given that in a recent EBMT study, identical LFS and RI were observed after 8 Gy and 12 Gy TBI-based conditioning [31].

Despite lower RI and superior LFS following TBI-based conditioning, OS was not significantly different among recipients of TBI as compared to both FluBu groups. This might be explained in part by a higher percentage of patients with Ph+ ALL in the two FluBu cohorts, given that the introduction of tyrosine kinase inhibitors (TKI) both into the induction therapy [32–35], and in the prophylaxis and treatment of relapse after alloHCT [36–38] has significantly improved the outcome of this patient subgroup. Unfortunately, available data among our patients were insufficient to estimate the influence of TKI given preemptively or for maintenance following alloHCT. A second reason for similar OS among the three cohorts could be a higher upper age limit among TBI recipients (76.1 vs 72.0 and 65.6 years). In the multivariate analysis, increasing age showed a significant negative impact on all outcome parameters, except RI, a finding in line with previous observations [9, 39].

Data on MRD before start of conditioning were available for about 74% of all patients. Among these, MRD positivity showed a significantly negative impact on RI and LFS. The evaluation of MRD as a predictive factor for post-transplant outcome was not the focus of our study, in particular considering the fact that MRD was examined according to local standards with different levels of sensitivity. Nevertheless, this finding is in line with other studies analyzing of the role of MRD status before alloHCT for outcome [20, 40, 41]. Importantly, MRD did not modify the role of TBI based conditioning on RI and LFS.

Some limitations of our study need to be considered. First, the reason why patients have been selected to receive their respective conditioning regimen could not be evaluated retrospectively. Further, the retrospective design was associated with several imbalances of some risk factors among the cohorts, which we tried to account for when fitting the multivariate models. Nevertheless, as discussed above, the lower percentage of Ph+ patients among TBI recipients might have counterbalanced the superior antileukemic effect of TBI. In contrast, imbalances concerning median age did not influence NRM as one may have expected. Similarly, different rates of in-vivo T-cell depletion (TCD) and differences concerning the year of transplantation among cohorts did not appear to significantly influence outcome. A deleterious effect of TCD on anti-tumor efficacy of chemotherapy-based RIC alloHCT had been observed in a large registry study [42]. However, only 4% of patients analyzed in that study suffered from ALL. In general, ALL is regarded as a disease with lower sensitivity to a graft-versus-leukemia effect [43]. Hence TCD might be less relevant among ALL patients as compared to myeloid diseases or slower proliferating lymphoid disorders.

In conclusion, this study represents the first direct comparison of intermediate intensity conditioning regimens comprising TBI versus chemotherapy in ALL patients >45 years. Considering the limits of a retrospective registry analysis, antileukemic efficacy was stronger after TBI-based conditioning within this cohort of 501 patients, as shown in the multivariate analysis by a lower RI and longer LFS. However, despite similar NRM rates, this only translated into a non-significant advantage in OS. Independently from the conditioning regimen, increasing age, MRD positivity and Ph+ ALL were the most important factors for overall outcome.

## Supplementary information

Supplementary Table 1

Supplementary Table 2

## Data Availability

Datasets of the analysis reported in this study may be available upon request via the corresponding author (CS).

## References

[1] Giebel S, Boumendil A, Labopin M, Seesaghur A, Baron F, Ciceri F (2019). Trends in the use of hematopoietic stem cell transplantation for adults with acute lymphoblastic leukemia in Europe: a report from the Acute Leukemia Working Party of the European Society for Blood and Marrow Transplantation (EBMT). Ann Hematol.

[2] Passweg JR, Baldomero H, Chabannon C, Basak GW, de la Camara R, Corbacioglu S (2021). Hematopoietic cell transplantation and cellular therapy survey of the EBMT: monitoring of activities and trends over 30 years. Bone Marrow Transplant.

[3] Eom KS, Shin SH, Yoon JH, Yahng SA, Lee SE, Cho BS (2013). Comparable long-term outcomes after reduced-intensity conditioning versus myeloablative conditioning allogeneic stem cell transplantation for adult high-risk acute lymphoblastic leukemia in complete remission. Am J Hematol.

[4] Dinmohamed AG, Szabó A, van der Mark M, Visser O, Sonneveld P, Cornelissen JJ (2016). Improved survival in adult patients with acute lymphoblastic leukemia in the Netherlands: a population-based study on treatment, trial participation and survival. Leukemia.

[5] Lennmyr E, Karlsson K, Ahlberg L, Garelius H, Hulegårdh E, Izarra AS (2019). Survival in adult acute lymphoblastic leukaemia (ALL): a report from the Swedish ALL Registry. Eur J Haematol.

[6] Marks DI, Forman SJ, Blume KG, Pérez WS, Weisdorf DJ, Keating A (2006). A comparison of cyclophosphamide and total body irradiation with etoposide and total body irradiation as conditioning regimens for patients undergoing sibling allografting for acute lymphoblastic leukemia in first or second complete remission. Biol Blood Marrow Transplant: J Am Soc Blood Marrow Transplant.

[7] Czyz A, Labopin M, Giebel S, Socié G, Apperley J, Volin L (2018). Cyclophosphamide versus etoposide in combination with total body irradiation as conditioning regimen for adult patients with Ph-negative acute lymphoblastic leukemia undergoing allogeneic stem cell transplant: on behalf of the ALWP of the European Society for Blood and Marrow Transplantation. Am J Hematol.

[8] Khimani F, Dutta M, Faramand R, Nishihori T, Perez AP, Dean E (2021). Impact of total body irradiation-based myeloablative conditioning regimens in patients with acute lymphoblastic leukemia undergoing allogeneic hematopoietic stem cell transplantation: systematic review and meta-analysis. Transplant Cell Ther.

[9] Mohty M, Labopin M, Volin L, Gratwohl A, Socié G, Esteve J (2010). Reduced-intensity versus conventional myeloablative conditioning allogeneic stem cell transplantation for patients with acute lymphoblastic leukemia: a retrospective study from the European Group for Blood and Marrow Transplantation. Blood.

[10] Roth-Guepin G, Canaani J, Ruggeri A, Labopin M, Finke J, Cornelissen JJ (2017). Allogeneic stem cell transplantation in acute lymphoblastic leukemia patients older than 60 years: a survey from the acute leukemia working party of EBMT. Oncotarget.

[11] Beelen DW, Bug G, Stelljes M, Gökbuget N, Arnold R. Durchführung der Stammzelltransplantation - Dosisadaptierte Konditionierungstherapie (46-55 Jahre). GMALL-Empfehlungen zur Stammzelltransplantation im Rahmen der Behandlung der akuten lymphatischen Leukämie der Erwachsenen. 2019;4.2:9.

[12] Hamaki T, Kami M, Kanda Y, Yuji K, Inamoto Y, Kishi Y (2005). Reduced-intensity stem-cell transplantation for adult acute lymphoblastic leukemia: a retrospective study of 33 patients. Bone Marrow Transplant.

[13] Ram R, Storb R, Sandmaier BM, Maloney DG, Woolfrey A, Flowers ME (2011). Non-myeloablative conditioning with allogeneic hematopoietic cell transplantation for the treatment of high-risk acute lymphoblastic leukemia. Haematologica.

[14] Rosko A, Wang HL, de Lima M, Sandmaier B, Khoury HJ, Artz A (2017). Reduced intensity conditioned allograft yields favorable survival for older adults with B-cell acute lymphoblastic leukemia. Am J Hematol.

[15] Lee SS, Jung SH, Do YR, Kim DS, Lee JH, Park HS (2020). Reduced-intensity conditioning with busulfan and fludarabine for allogeneic hematopoietic stem cell transplantation in acute lymphoblastic leukemia. Yonsei Med J.

[16] Peric Z, Labopin M, Peczynski C, Polge E, Cornelissen J, Carpenter B (2020). Comparison of reduced-intensity conditioning regimens in patients with acute lymphoblastic leukemia >45 years undergoing allogeneic stem cell transplantation-a retrospective study by the Acute Leukemia Working Party of EBMT. Bone Marrow Transplant.

[17] Saraceni F, Scortechini I, Fiorentini A, Dubbini MV, Mancini G, Federici I (2021). Conditioning regimens for frail patients with acute leukemia undergoing allogeneic stem cell transplant: how to strike gently. Clin Hematol Int.

[18] Filipovich AH, Weisdorf D, Pavletic S, Socie G, Wingard JR, Lee SJ (2005). National Institutes of Health consensus development project on criteria for clinical trials in chronic graft-versus-host disease: I. Diagnosis and staging working group report. Biol Blood Marrow Transplant: J Am Soc Blood Marrow Transplant.

[19] Jagasia MH, Greinix HT, Arora M, Williams KM, Wolff D, Cowen EW (2015). National institutes of health consensus development project on criteria for clinical trials in chronic graft-versus-host disease: I. the 2014 diagnosis and staging working group report. Biol Blood Marrow Transplant: J Am Soc Blood Marrow Transplant.

[20] Pavlů J, Labopin M, Niittyvuopio R, Socié G, Yakoub-Agha I, Wu D (2019). Measurable residual disease at myeloablative allogeneic transplantation in adults with acute lymphoblastic leukemia: a retrospective registry study on 2780 patients from the acute leukemia working party of the EBMT. J Hematol Oncol.

[21] Abdelaty MM, Gawaly A, Fathy GM, Kabbash I, Taha A (2020). Irradiation free conditioning regimen is associated with high relapse rate in Egyptian patients with acute lymphoblastic leukemia following allogeneic hematopoietic stem cell transplantation. J Egypt Natl Cancer Inst.

[22] Marks DI, Stelljes M. Acute Lymphoblastic Leukemia in Adults. In: Enric Carreras CD, Mohamad M, N Kröger (ed.) The EBMT Handbook. Springer Open, 2019, pp 531–8. 32091765

[23] Peters C, Dalle JH, Locatelli F, Poetschger U, Sedlacek P, Buechner J (2021). Total body irradiation or chemotherapy conditioning in childhood ALL: a multinational, randomized, noninferiority phase III study. J Clin Oncol: Off J Am Soc Clin Oncol.

[24] Akahoshi Y, Nishiwaki S, Arai Y, Harada K, Najima Y, Kanda Y (2020). Reduced-intensity conditioning is a reasonable alternative for Philadelphia chromosome-positive acute lymphoblastic leukemia among elderly patients who have achieved negative minimal residual disease: a report from the Adult Acute Lymphoblastic Leukemia Working Group of the JSHCT. Bone Marrow Transplant.

[25] Gao RW, Weisdorf DJ, DeFor TE, Ehler E, Dusenbery KE (2019). Influence of total body irradiation dose rate on idiopathic pneumonia syndrome in acute leukemia patients undergoing allogeneic hematopoietic cell transplantation. Int J Radiat Oncol Biol Phys.

[26] Vogel J, Hui S, Hua CH, Dusenbery K, Rassiah P, Kalapurakal J (2021). Pulmonary toxicity after total body irradiation - critical review of the literature and recommendations for toxicity reporting. Front Oncol.

[27] Oertel M, Martel J, Mikesch JH, Scobioala S, Reicherts C, Kröger K (2021). The burden of survivorship on hematological patients-long-term analysis of toxicities after total body irradiation and allogeneic stem cell transplantation. Cancers.

[28] Onizuka M, Fujii N, Nakasone H, Ogata M, Atsuta Y, Suzuki R (2022). Risk factors and prognosis of non-infectious pulmonary complications after allogeneic hematopoietic stem cell transplantation. Int J Hematol.

[29] Giebel S, Labopin M, Sobczyk-Kruszelnicka M, Stelljes M, Byrne JL, Fegueux N (2021). Total body irradiation + fludarabine compared to busulfan + fludarabine as “reduced-toxicity conditioning” for patients with acute myeloid leukemia treated with allogeneic hematopoietic cell transplantation in first complete remission: a study by the Acute Leukemia Working Party of the EBMT. Bone Marrow Transplant.

[30] Zhang H, Fan Z, Huang F, Han L, Xu Y, Xu N (2023). Busulfan plus cyclophosphamide versus total body irradiation plus cyclophosphamide for adults acute B lymphoblastic leukemia: an open-label, multicenter, phase III trial. J Clin Oncol: Off J Am Soc Clin Oncol.

[31] Spyridonidis A, Labopin M, Savani B, Giebel S, Bug G, Schönland S (2023). Reduced 8-gray compared to standard 12-gray total body irradiation for allogeneic transplantation in first remission acute lymphoblastic leukemia: a study of the acute leukemia working party of the EBMT. HemaSphere.

[32] Kim DY, Joo YD, Lim SN, Kim SD, Lee JH, Lee JH (2015). Nilotinib combined with multiagent chemotherapy for newly diagnosed Philadelphia-positive acute lymphoblastic leukemia. Blood.

[33] Jabbour E, Kantarjian H, Ravandi F, Thomas D, Huang X, Faderl S (2015). Combination of hyper-CVAD with ponatinib as first-line therapy for patients with Philadelphia chromosome-positive acute lymphoblastic leukaemia: a single-centre, phase 2 study. Lancet Oncol.

[34] Ravandi F, Othus M, O’Brien SM, Forman SJ, Ha CS, Wong JYC (2016). US intergroup study of chemotherapy plus dasatinib and allogeneic stem cell transplant in philadelphia chromosome positive ALL. Blood Adv.

[35] Chiaretti S, Vitale A, Vignetti M, Piciocchi A, Fazi P, Elia L (2016). A sequential approach with imatinib, chemotherapy and transplant for adult Ph+ acute lymphoblastic leukemia: final results of the GIMEMA LAL 0904 study. Haematologica.

[36] Giebel S, Czyz A, Ottmann O, Baron F, Brissot E, Ciceri F, et al. Use of tyrosine kinase inhibitors to prevent relapse after allogeneic hematopoietic stem cell transplantation for patients with Philadelphia chromosome-positive acute lymphoblastic leukemia: a position statement of the Acute Leukemia Working Party of the European Society for Blood and Marrow Transplantation. Cancer. 2016;122:2941–51. 10.1002/cncr.30130 27309127

[37] Hirschbühl K, Labopin M, Houhou M, Gabellier L, Labussière-Wallet H, Lioure B (2021). Second- and third-generation tyrosine kinase inhibitors for Philadelphia-positive adult acute lymphoblastic leukemia relapsing post allogeneic stem cell transplantation-a registry study on behalf of the EBMT Acute Leukemia Working Party. Bone Marrow Transplant.

[38] Bazarbachi A, Labopin M, Aljurf M, Niittyvuopio R, Balsat M, Blaise D, et al. 20-year steady increase in survival of adult patients with relapsed philadelphia-positive acute lymphoblastic leukemia post allogeneic hematopoietic cell transplantation. Clin Cancer Res. 2022;28:1004–12. 10.1158/1078-0432.CCR-21-2675 35022319

[39] Leonard JT, Hayes-Lattin B (2018). Reduced intensity conditioning allogeneic hematopoietic stem cell transplantation for acute lymphoblastic leukemia; current evidence, and improving outcomes going forward. Curr Hematol Malig Rep.

[40] Esteve J, Giebel S, Labopin M, Czerw T, Wu D, Volin L (2021). Allogeneic hematopoietic stem cell transplantation for adult patients with t(4;11)(q21;q23) KMT2A/AFF1 B-cell precursor acute lymphoblastic leukemia in first complete remission: impact of pretransplant measurable residual disease (MRD) status. An analysis from the Acute Leukemia Working Party of the EBMT. Leukemia.

[41] Fernando F, Robertson HF, El-Zahab S, Pavlů J (2021). How I use measurable residual disease in the clinical management of adult acute lymphoblastic leukemia. Clin Hematol Int.

[42] Soiffer RJ, Lerademacher J, Ho V, Kan F, Artz A, Champlin RE (2011). Impact of immune modulation with anti-T-cell antibodies on the outcome of reduced-intensity allogeneic hematopoietic stem cell transplantation for hematologic malignancies. Blood.

[43] Alyea EP, DeAngelo DJ, Moldrem J, Pagel JM, Przepiorka D, Sadelin M (2010). NCI first international workshop on the biology, prevention and treatment of relapse after allogeneic hematopoietic cell transplantation: report from the committee on prevention of relapse following allogeneic cell transplantation for hematologic malignancies. Biol Blood Marrow Transplant: J Am Soc Blood Marrow Transplant.

