# Supplementary material for: Total body irradiation versus busulfan based intermediate intensity conditioning for stem cell transplantation in ALL patients >45 years—a registry-based study by the Acute Leukemia Working Party of the EBMT
Source: Bone Marrow Transplant. 2023 May 5;58(8):874–80. doi: 10.1038/s41409-023-01966-w (PMC10400409; doi:10.1038/s41409-023-01966-w)
Supplement: Supplementary file 1 — Supplementary Table 1 [file 41409_2023_1966_MOESM1_ESM.docx]

**Supplementary Table 1:** Univariate analysis of conditioning on outcome parameters

|  | **RI** | **NRM** | **LFS** | **OS** | **GRFS** | **aGVHD II-IV** | **aGVHD III-IV** | **cGVHD** | **ext. cGVHD** |
| --- | --- | --- | --- | --- | --- | --- | --- | --- | --- |
| **FluTBI8** | 24.7%[18.9-31] | 17.3%[12.3-23] | 58%[50.5-64.7] | 68.5%[61.1-74.7] | 39.9%[32.8-46.9] | 26.3%[21.1-31.8] | 9%[5.9-13] | 45.7%[38.5-52.7] | 23.5%[17.7-29.8] |
| **FluBu6.4** | 37.3%[30.1-44.4] | 20.1%[14.5-26.3] | 42.7%[35.2-49.9] | 57%[49.2-64.1] | 34.3%[27.3-41.4] | 26.3%[20.1-32.8] | 8.8%[5.3-13.5] | 33.2%[26.1-40.5] | 15.9%[10.8-22] |
| **FluBu9.6** | 30.9%[18.4-44.4] | 24%[13.2-36.7] | 45%[30.7-58.4] | 62.2%[46.7-74.4] | 40.1%[26.3-53.6] | 29.2%[17-42.4] | 8.2%[2.6-18] | 31.1%[18.1-45] | 10.9%[3.9-22] |
| **p value (global)** | 0.014 | 0.38 | 0.003 | 0.06 | 0.29 | 0.9 | 0.97 | 0.018 | 0.07 |

*RI* relapse incidence, *NRM* non-relapse mortality, *LFS* leukemia-free survival, *OS* overall survival, *GRFS* graft-versus-host disease-free/ relapse-free survival, *aGvHD* acute graft-versus-host disease, *cGvHD* chronic graft-versus-host disease
