# Supplementary material for: Total body irradiation versus busulfan based intermediate intensity conditioning for stem cell transplantation in ALL patients >45 years—a registry-based study by the Acute Leukemia Working Party of the EBMT
Source: Bone Marrow Transplant. 2023 May 5;58(8):874–80. doi: 10.1038/s41409-023-01966-w (PMC10400409; doi:10.1038/s41409-023-01966-w)
Supplement: Supplementary file 2 — Supplementary Table 2 [file 41409_2023_1966_MOESM2_ESM.docx]

**Supplementary Table 2:** Multivariate analysis of risk factors on graft-versus-host disease

|  | **ACUTE GVHD II-IV** | | **ACUTE GVHD III-IV** | | **chronic GVHD** | | **extensive chronic GVHD** | |
| --- | --- | --- | --- | --- | --- | --- | --- | --- |
|  | **HR (95% CI)** | **p value** | **HR (95% CI)** | **p value** | **HR (95% CI)** | **p value** | **HR (95% CI)** | **p value** |
| **FluTBI8 (ref.)** | 1 |  | 1 |  | 1 |  | 1 |  |
| **FluBu6.4** | 1 (0.62-1.6) | 1 | 0.99 (0.45-2.2) | 0.98 | 0.87 (0.58-1.31) | 0.51 | 1.04 (0.54-1.99) | 0.92 |
| **FluBu9.6** | 1.21 (0.65-2.25) | 0.55 | 0.91 (0.3-2.73) | 0.86 | 0.8 (0.45-1.44) | 0.46 | 0.74 (0.27-2.04) | 0.56 |
| **Age (per 10y)** | 1.01 (0.74-1.39) | 0.93 | 1.34 (0.76-2.36) | 0.31 | 1.09 (0.81-1.47) | 0.57 | 1.01 (0.65-1.57) | 0.96 |
| **Ph neg B-ALL (ref.)** | 1 |  | 1 |  | 1 |  | 1 |  |
| **Ph pos B-ALL** | 1.13 (0.71-1.79) | 0.61 | 1.5 (0.6-3.75) | 0.38 | 0.72 (0.48-1.08) | 0.11 | 1.09 (0.59-2.01) | 0.79 |
| **T-ALL** | 1.29 (0.69-2.39) | 0.42 | 2.39 (0.83-6.86) | 0.11 | 0.87 (0.49-1.55) | 0.63 | 0.63 (0.24-1.7) | 0.36 |
| **MRD - (ref.)** | 1 |  | 1 |  | 1 |  | 1 |  |
| **MRD +** | 0.95 (0.62-1.46) | 0.82 | 2.23 (1.1-4.53) | 0.026 | 0.94 (0.63-1.39) | 0.74 | 0.87 (0.48-1.58) | 0.65 |
| **MRD unknown** | 1.04 (0.63-1.71) | 0.88 | 1.03 (0.39-2.67) | 0.96 | 0.94 (0.62-1.42) | 0.77 | 0.89 (0.46-1.73) | 0.74 |
| **Year of HCT** | 1 (0.94-1.06) | 0.97 | 0.98 (0.9-1.08) | 0.73 | 0.97 (0.92-1.02) | 0.24 | 0.99 (0.91-1.07) | 0.81 |
| **Female to Male** | 0.72 (0.41-1.27) | 0.26 | 1.17 (0.5-2.74) | 0.72 | 0.96 (0.63-1.48) | 0.87 | 1.15 (0.62-2.16) | 0.65 |
| **KPS>=90** | 1 (0.67-1.49) | 0.99 | 1.45 (0.7-2.99) | 0.32 | 1.29 (0.9-1.85) | 0.16 | 1.02 (0.6-1.73) | 0.94 |
| **MSD (ref.)** | 1 |  | 1 |  | 1 |  | 1 |  |
| **UD 10/10** | 1.23 (0.79-1.91) | 0.37 | 0.77 (0.36-1.62) | 0.49 | 1.13 (0.76-1.69) | 0.54 | 0.99 (0.55-1.79) | 0.97 |
| **UD 9/10** | 1.62 (0.9-2.92) | 0.11 | 0.56 (0.16-1.97) | 0.37 | 1.89 (1.15-3.1) | 0.012 | 1.54 (0.7-3.4) | 0.28 |
| **PB vs BM** | 1.14 (0.52-2.53) | 0.74 | 1.5 (0.35-6.42) | 0.58 | 1.11 (0.58-2.14) | 0.75 | 0.84 (0.34-2.09) | 0.7 |
| **in vivo TCD** | 1.08 (0.65-1.82) | 0.76 | 0.84 (0.37-1.9) | 0.68 | 0.45 (0.29-0.68) | 0,0001 | 0.4 (0.21-0.77) | 0.006 |
| **Patient/Donor CMV -/- vs other** | 1.27 (0.86-1.89) | 0.23 | 1.48 (0.75-2.92) | 0.25 | 1.32 (0.92-1.91) | 0.14 | 1.74 (1.03-2.94) | 0.038 |
| **Centre (frailty)** |  | 0.18 |  | 0.94 |  | 0.23 |  | 0.15 |

*HR* hazard ratio, *CI* confidence interval, *ref.* reference, *y* year, *Ph* Philadelphia chromosome, *MRD* measurable residual disease, *HCT* hematopoietic cell transplantation, *KPS* Karnofsky performance status, *MSD* matched sibling donor, *UD* unrelated donor, *PB* peripheral blood, *BM* bone marrow, *TCD* T-cell depletion, *CMV* cytomegalovirus.
